# Crystal structure of *N*′-[2-(benzo[*d*]thia­zol-2-yl)acet­yl]benzohydrazide, an achiral compound crystallizing in space group *P*1 with *Z* = 1

**DOI:** 10.1107/S2056989021007672

**Published:** 2021-08-03

**Authors:** Rasha A. Azzam, Galal H. Elgemeie, Mona M. Seif, Peter G. Jones

**Affiliations:** a Chemistry Department, Faculty of Science, Helwan University, Cairo, Egypt; b Institut für Anorganische und Analytische Chemie, Technische Universität Braunschweig, Hagenring 30, D-38106 Braunschweig, Germany

**Keywords:** thia­zole, hydrazide, hydrogen bond, space group *P*1, crystal structure

## Abstract

In the mol­ecule of the title compound, C_16_H_13_N_3_O_2_S, one hydrazinic nitro­gen atom is essentially planar, but the other is slightly pyramidalized. The torsion angle about the hydrazinic bond is 66.44 (15)°. Both hydrazinic hydrogen atoms lie anti­periplanar to the oxygen of the adjacent carbonyl group. The mol­ecular packing is a layer structure determined by two classical hydrogen bonds, N—H⋯O=C and N—H⋯N_thia­zole_. The space group is *P*1 with *Z* = 1, which is unusual for an achiral organic compound.

## Abstract 2

The title compound crystallizes in space group *P*1 despite being achiral. Two classical hydrogen bonds link the mol­ecules to form a layer structure.

## Chemical context   

Heterocycles represent a link between organic synthesis and pharmaceutical chemistry, thereby encouraging researchers to discover new hetereocyclic drug candidates. One of the most prominent heterocycles is benzo­thia­zole, a privileged scaffold in the field of synthetic and medicinal chemistry (Elgemeie *et al.*, 2000*a* ▸,*b* ▸). Its derivatives and metal complexes possess a wide range of pharmacological properties and a high degree of structural diversity that have proved vital for the investigation for novel therapeutics (Elgemeie *et al.*, 2020 ▸; Gill *et al.*, 2015 ▸). The carbon atom C2 (standard numbering; the carbon atom between nitro­gen and sulfur) is the most attractive site both from a synthetic and medicinal point of view (Azzam *et al.*, 2020*a* ▸,*b* ▸). As structure–activity relationships have shown, changes in the substituent at C2 can induce marked changes in the biological activity (Azzam *et al.*, 2017*a* ▸,*b* ▸). Numerous biologically potent mol­ecules containing 2-substituted benzo­thia­zole scaffolds have extensive biological applications (Keri *et al.*, 2015 ▸), such as anti-microbial (König *et al.*, 2011 ▸), anti-malarial (Bowyer *et al.*, 2007 ▸) and anti-inflammatory (Wang *et al.*, 2009 ▸). Among the 2-substituted benzo­thia­zoles, 2-aryl benzo­thia­zoles are versatile scaffolds that have major biological and industrial applications (Kamal *et al.*, 2011 ▸). Part of our research has therefore concentrated on the synthetic pathways of 2-aryl­benzo­thia­zoles (Azzam *et al.*, 2019 ▸; Elgemeie & Elghandour, 1990 ▸). Recently, we contributed to current progress in the manufacturing and biological estimation of 2-aryl, 2-pyridyl and 2-pyrimidylbenzo­thia­zoles and other anti­metabolites as potent chemotherapeutic agents (Azzam *et al.*, 2020*c* ▸; Metwally *et al.*, 2021 ▸). Here we deal with synthetic approaches to the new compound *N*′-(2-(benzo[*d*]thia­zol-2-yl)acet­yl)benzohydrazide (**3**). Compound **3** was prepared by the reaction of 2-(benzo[*d*]thia­zol-2-yl)acetohydrazide (**2**) with benzoyl chloride in the presence of pyridine at room temperature. The structure of **3** was initially determined on the basis of spectroscopic data and elemental analysis. In order to establish the structure of the product unambiguously, its crystal structure was determined and is presented here.
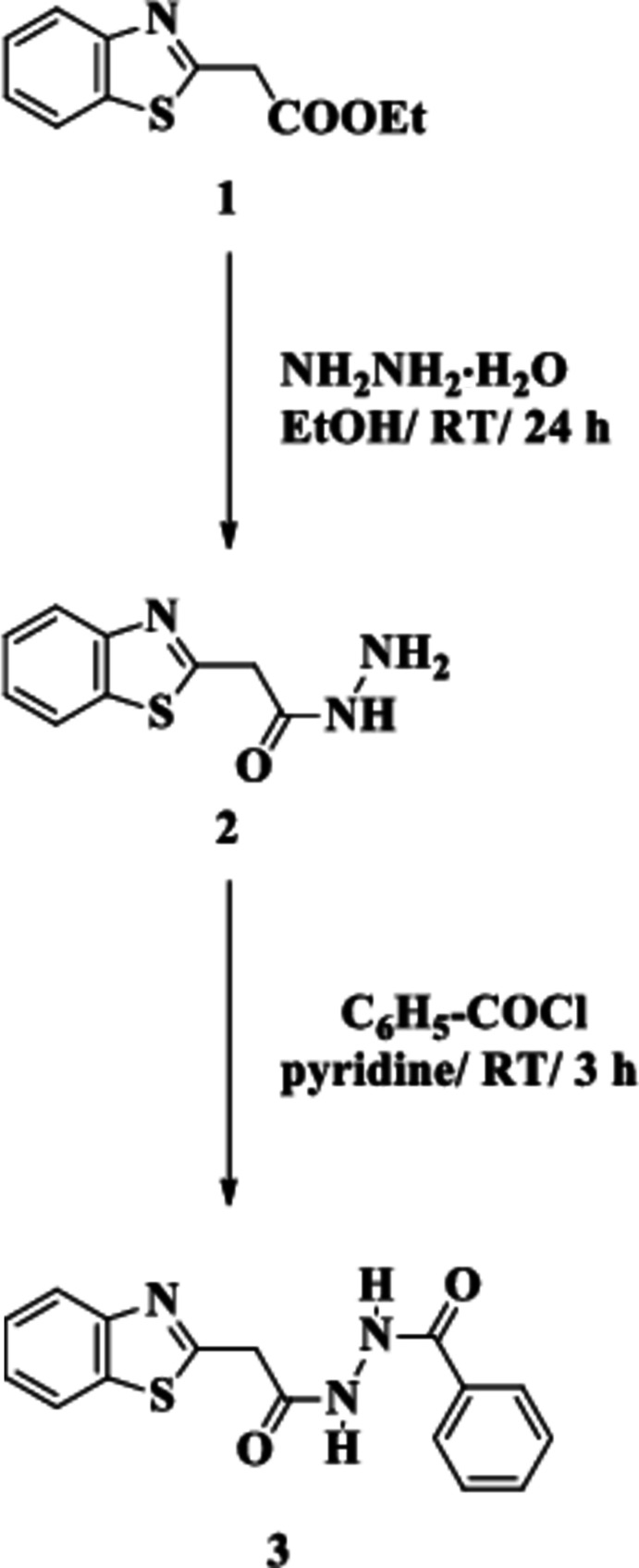


## Structural commentary   

**Figure 1:**
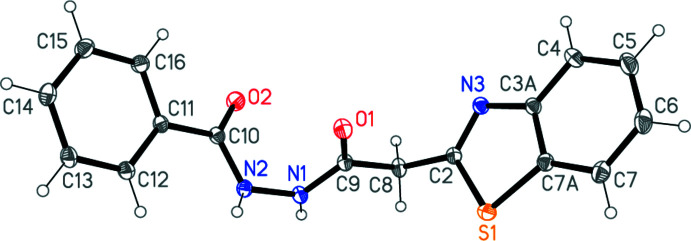
The mol­ecule of compound **3** in the crystal. Ellipsoids represent 50% probability levels.

The structure determination confirms the formation of compound **3** (Fig. 1 ▸). Bond lengths and angles may be regarded as normal (Allen *et al.*, 1987 ▸); a selection is presented in Table 1 ▸. The geometry at the hydrazinic nitro­gen atom N1 is essentially planar, but N2 is slightly pyramidalized [angle sum 355 (2)°; the nitro­gen atom lies 0.15 (1) Å out of the plane of its substituents]. The general shape of the mol­ecule is defined by the torsion angles along the atom chain S1—C2—C8—C9—N1—N2—C10—C11—C12, which are also given in Table 1 ▸; in particular, the torsion angle about the hydrazine N1—N2 bond is 66.44 (15)° [*cf*. H01—N1—N2—H02 101 (3)°]. Each hydrazinic hydrogen atom lies anti­periplanar to a carbonyl oxygen atom across the respective N—C bond. The inter­planar angle between the benzo­thia­zol group and the phenyl ring is 75.65 (3)°.

## Supra­molecular features   

**Figure 2:**
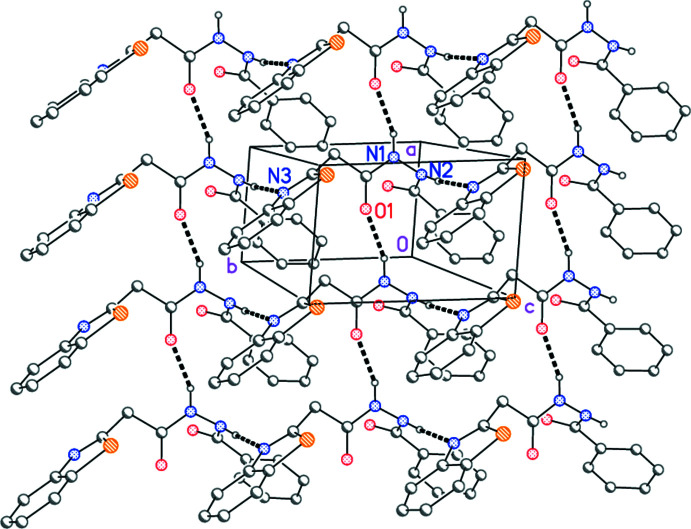
Packing diagram of compound **3** viewed perpendicular to the *ab* plane. Dashed lines represent classical hydrogen bonds. Hydrogen atoms not involved in hydrogen bonding are omitted for clarity. Selected atoms of the asymmetric unit are labelled.

Two classical hydrogen bonds, from the hydrazinic hydrogen atoms to the carbonyl oxygen O1 and the heterocyclic nitro­gen N3 (Table 2 ▸), link the mol­ecules to form layers parallel to the *ab* plane (Fig. 2 ▸).

## Database survey   

A database search (CSD Version 5.41) for other structures containing the same benzo­thia­zol-acetyl­hydrazide moiety gave only one hit, refcode JEBQOZ, with a *p*-tosyl­ate group replacing the benzoyl group of **3**; this was our previous publication (Azzam *et al.*, 2017*b* ▸). There are major conformational differences between the two structures, *e.g*. the C—C—C(=O)—N torsion angle of JEBQOZ is −109.79 (19)° in contrast to −152.41 (11)° in the title structure. The average database bond lengths C2—S and C2—N for the benzo[*d*]thia­zole ring system were calculated; for 444 hits (600 different mol­ecules) the values were 1.750 (16) and 1.300 (29) Å, respectively, virtually unchanged from the values we obtained previously (Azzam *et al.*, 2017*b* ▸); however, we regret having mistyped the latter value as 1.200.

Anecdotal evidence, combined with previous experience, would suggest that it is unusual for an achiral compound to crystallize in space group *P*1, which may be considered as a moderately rare space group; of the over 1.1 million structures in the Cambridge database, only 9843 are in *P*1 (8832 with coordinates available, 6730 of these without disorder).

We therefore wished to see how many of the *P*1 structures in the CSD, particularly those with *Z* = 1, were achiral. Unfortunately, there is at present no means of identifying, labelling and searching for chirality or chiral (‘asymmetric’) atoms using the standard *ConQuest* search routines, and it is clearly unfeasible to check all the *P*1 structures by hand. We therefore began by simply considering the small and possibly non-representative subset of 20 *P*1 structures (13 with *Z* > 1) that were determined by PGJ. Of these, 14 were pure enanti­omers; for 12 of these, the absolute configuration was determined. Of the remaining six, five were not organic compounds [two metal complexes with *Z* = 1 (Jones *et al.*, 1996 ▸; Filimon *et al.*, 2014 ▸), two organotellurium compounds (Jones *et al.*, 2015 ▸, *Z* = 1; du Mont *et al.*, 2010 ▸, *Z* = 4), and one phosphane sulfide (Taouss & Jones, 2013 ▸, *Z* = 2)], and the remaining structure (Focken *et al.*, 2001 ▸, *Z* = 4) displayed planar chirality, but contained no ‘asymmetric’ atom. On this limited basis, we would therefore postulate that is very rare for achiral organic compounds to crystallize in *P*1, especially with *Z* = 1. An extension of this survey to all *P*1 structures in the CCDC is being planned.

## Synthesis and crystallization   

A mixture of 2-(benzo[*d*]thia­zol-2-yl)acetohydrazide **2** (0.08 mol) and pyridine (10 mL) was stirred for 15 min at room temperature. Benzoyl chloride (0.16 mol) was then added gradually to the reaction mixture, which was stirred for 15 min at 273 K. The reaction mixture was left to stand at room temperature for another 3 h, then poured onto ice water and neutralized with HCl. The precipitate thus formed was filtered off and dried to produce a white solid product **3**. This was washed with ethyl acetate and recrystallized from ethanol; yield 85%, m.p. 487 K.

IR (KBr, cm^−1^): υ 3429–3284 (NH), 2974 (CH aromatic), 1696, 1662 (2CO); ^1^H NMR (400 MHz, DMSO-*d*_6_): δ 4.23 (*s*, 2H, CH_2_), 7.43 (*t*, *J* = 7.2 Hz, 1H, benzo­thia­zole H), 7.49–7.53 (*m*, 3H, C_6_H_5_), 7.58 (*t*, *J* = 7.2 Hz, 1H, benzo­thia­zole H), 7.91 (*d*, *J* = 7.2 Hz, 2H, C_6_H_5_), 7.99 (*d*, *J* = 9.6 Hz, 1H, benzo­thia­zole H), 8.09 (*d*, *J* = 9.2 Hz, 1H, benzo­thia­zole H), 10.48 (*s*, 1H, NH), 10.55 (*s*, 1H, NH); ^13^C NMR (100 MHz, DMSO-*d*_6_): δ 39.4 (CH_2_), 122.5, 122.8, 125.5, 126.5, 127.9, 128.9, 132.4, 132.8, 136.9, 152.7, 165.0 (Ar-C), 166.0, 167.1 (2CO). Analysis: calculated for C_16_H_13_N_3_O_2_S (311.36): C 61.72; H 4.21; N 13.50%; found: C 61.70; H 4.22; N 13.55%.

## Refinement   

Crystal data, data collection and structure refinement details are summarized in Table 3 ▸. The hydrogen atoms of the NH groups were refined freely. Other hydrogens were included using a riding model starting from calculated positions (C—H_aromatic_ = 0.95, C—H_methyl­ene_ = 0.99 Å). The *U*(H) values were fixed at 1.2 times the equivalent *U*_iso_ value of the parent carbon atoms.

The compound contains no chiral centres and crystallizes only by chance in a chiral (Sohncke) space group.

## Supplementary Material

Crystal structure: contains datablock(s) I, global. DOI: 10.1107/S2056989021007672/yk2155sup1.cif

Structure factors: contains datablock(s) I. DOI: 10.1107/S2056989021007672/yk2155Isup2.hkl

Supporting information file. DOI: 10.1107/S2056989021007672/yk2155Isup3.cml

CCDC reference: 2099652

Additional supporting information:  crystallographic information; 3D view; checkCIF report

## Figures and Tables

**Table 1:** Selected geometric parameters (Å, °)

S1—C7*A*	1.7310 (13)	N1—N2	1.3901 (14)
S1—C2	1.7422 (12)	N3—C3*A*	1.3939 (16)
C2—N3	1.2993 (16)		
			
C7*A*—S1—C2	89.49 (6)	C10—N2—N1	117.28 (10)
N3—C2—S1	115.83 (9)	C2—N3—C3*A*	110.61 (10)
C9—N1—N2	119.00 (10)		
			
C9—N1—N2—C10	66.44 (15)	N2—C10—C11—C12	−18.46 (17)
S1—C2—C8—C9	80.26 (12)	O1—C9—N1—H01	175 (2)
N2—N1—C9—C8	−173.21 (10)	O2—C10—N2—H02	166 (2)
C2—C8—C9—N1	−152.41 (11)	H01—N1—N2—H02	101 (3)
N1—N2—C10—C11	−167.79 (10)		

**Table 2:** Hydrogen-bond geometry (Å, °)

*D*—H⋯*A*	*D*—H	H⋯*A*	*D*⋯*A*	*D*—H⋯*A*
N1—H01⋯O1^i^	0.88 (3)	2.02 (3)	2.8438 (14)	157 (3)
N2—H02⋯N3^ii^	0.85 (3)	2.15 (3)	2.9736 (15)	162 (3)

Symmetry codes: (i) x+1, y, z; (ii) x, y-1, z.

**Table 3:** Experimental details

Crystal data	
Chemical formula	C_16_H_13_N_3_O_2_S
*M*_r_	311.35
Crystal system, space group	Triclinic, *P*1
Temperature (K)	100
*a*, *b*, *c* (Å)	4.71248 (9), 6.96463 (14), 11.5455 (3)
α, β, γ (°)	105.6168 (18), 95.7876 (16), 95.9993 (16)
*V* (Å^3^)	359.64 (1)
*Z*	1
Radiation type	Mo *K*α
μ (mm^−1^)	0.24
Crystal size (mm)	0.20 × 0.16 × 0.05

Data collection	
Diffractometer	XtaLAB Synergy, HyPix
Absorption correction	Multi-scan (*CrysAlis PRO*; Rigaku OD, 2020 ▸)
*T*_min_, *T*_max_	0.844, 1.000
No. of measured, independent and observed [*I* > 2σ(*I*)] reflections	60895, 6522, 6312
*R*_int_	0.034
(sin θ/λ)_max_ (Å^−1^)	0.843

Refinement	
*R*[*F*^2^ > 2σ(*F* ^2^)], *wR*(*F* ^2^), *S*	0.030, 0.078, 1.06
No. of reflections	6522
No. of parameters	207
No. of restraints	3
H-atom treatment	H atoms treated by a mixture of independent and constrained refinement
Δρ_max_, Δρ_min_ (e Å^−3^)	0.41, −0.27
Absolute structure	Flack *x* determined using 2959 quotients [(*I* ^+^)−(*I* ^−^)]/[(*I* ^+^)+(*I* ^−^)] (Parsons *et al.*, 2013 ▸)
Absolute structure parameter	−0.016 (12)

Computer programs: *CrysAlis PRO* (Rigaku OD, 2020 ▸), *SHELXT* (Sheldrick, 2015*a* ▸), *SHELXL2018/3* (Sheldrick, 2015*b* ▸) and *XP* (Siemens, 1994 ▸).

## supplementary crystallographic information

### Crystal data

**Table:**

C_16_H_13_N_3_O_2_S	*Z* = 1
*M**_r_* = 311.35	*F*(000) = 162
Triclinic, *P*1	*D*_x_ = 1.438 Mg m^−^^3^
*a* = 4.71248 (9) Å	Mo *K*α radiation, λ = 0.71073 Å
*b* = 6.96463 (14) Å	Cell parameters from 40070 reflections
*c* = 11.5455 (3) Å	θ = 3.0–37.0°
α = 105.6168 (18)°	µ = 0.24 mm^−^^1^
β = 95.7876 (16)°	*T* = 100 K
γ = 95.9993 (16)°	Plate, colourless
*V* = 359.64 (1) Å^3^	0.20 × 0.16 × 0.05 mm

### Data collection

**Table:**

XtaLAB Synergy, HyPix diffractometer	6522 independent reflections
Radiation source: micro-focus sealed X-ray tube, PhotonJet (Mo) X-ray Source	6312 reflections with *I* > 2σ(*I*)
Mirror monochromator	*R*_int_ = 0.034
Detector resolution: 10.0000 pixels mm^-1^	θ_max_ = 36.8°, θ_min_ = 3.1°
ω scans	*h* = −7→7
Absorption correction: multi-scan (CrysAlisPro; Rigaku OD, 2020)	*k* = −11→11
*T*_min_ = 0.844, *T*_max_ = 1.000	*l* = −18→19
60895 measured reflections	

### Refinement

**Table:**

Refinement on *F*^2^	Hydrogen site location: mixed
Least-squares matrix: full	H atoms treated by a mixture of independent and constrained refinement
*R*[*F*^2^ > 2σ(*F*^2^)] = 0.030	*w* = 1/[σ^2^(*F*_o_^2^) + (0.0486*P*)^2^ + 0.0499*P*] where *P* = (*F*_o_^2^ + 2*F*_c_^2^)/3
*wR*(*F*^2^) = 0.078	(Δ/σ)_max_ < 0.001
*S* = 1.06	Δρ_max_ = 0.41 e Å^−^^3^
6522 reflections	Δρ_min_ = −0.27 e Å^−^^3^
207 parameters	Absolute structure: Flack *x* determined using 2959 quotients [(*I*^+^)-(*I*^-^)]/[(*I*^+^)+(*I*^-^)] (Parsons *et al.*, 2013)
3 restraints	Absolute structure parameter: −0.016 (12)
Primary atom site location: structure-invariant direct methods	

### Special details

**Table:**

Geometry. All esds (except the esd in the dihedral angle between two l.s. planes) are estimated using the full covariance matrix. The cell esds are taken into account individually in the estimation of esds in distances, angles and torsion angles; correlations between esds in cell parameters are only used when they are defined by crystal symmetry. An approximate (isotropic) treatment of cell esds is used for estimating esds involving l.s. planes. Least-squares planes (x,y,z in crystal coordinates) and deviations from them (* indicates atom used to define plane) 3.5462 (0.0009) x + 3.6978 (0.0019) y - 0.2128 (0.0045) z = 6.1650 (0.0025) * -0.0069 (0.0007) S1 * 0.0023 (0.0009) C2 * 0.0044 (0.0009) N3 * -0.0011 (0.0011) C4 * -0.0046 (0.0012) C5 * 0.0007 (0.0012) C6 * 0.0082 (0.0011) C7 * -0.0004 (0.0011) C3A * -0.0026 (0.0011) C7A -0.0318 (0.0015) C8 Rms deviation of fitted atoms = 0.0043 - 3.3343 (0.0019) x + 1.8849 (0.0037) y + 7.6563 (0.0052) z = 0.2749 (0.0019) Angle to previous plane (with approximate esd) = 75.652 ( 0.033 ) * 0.0041 (0.0009) C11 * 0.0008 (0.0009) C12 * -0.0051 (0.0009) C13 * 0.0044 (0.0010) C14 * 0.0006 (0.0010) C15 * -0.0049 (0.0009) C16 -0.0026 (0.0020) C10 Rms deviation of fitted atoms = 0.0038 ============================================================================= - 0.0418 (0.0830) x - 3.0734 (0.0043) y + 11.3227 (0.0146) z = 3.7743 (0.0598) * 0.0000 (0.0000) N2 * 0.0000 (0.0001) C9 * 0.0000 (0.0000) H01 0.0634 (0.0123) N1 Rms deviation of fitted atoms = 0.0000 4.0751 (0.0123) x - 3.1432 (0.0992) y - 3.6530 (0.1475) z = 1.0965 (0.0176) * 0.0000 (0.0001) N1 * 0.0000 (0.0001) C10 * 0.0000 (0.0000) H02 0.1480 (0.0122) N2 Rms deviation of fitted atoms = 0.0000 ============================================================================= Further torsion angles: 97.53 ( 0.11) O2 - C10 ··· C9 - O1 175.44 ( 2.18) H01 - N1 - C9 - O1 165.89 ( 2.18) H02 - N2 - C10 - O2 101.08 ( 2.76) H01 - N1 - N2 - H02

### Fractional atomic coordinates and isotropic or equivalent isotropic displacement parameters (Å^2^)

**Table:**

	*x*	*y*	*z*	*U*_iso_*/*U*_eq_	
S1	0.88408 (5)	0.86164 (4)	0.76703 (3)	0.01738 (7)	
C2	0.8642 (3)	0.87459 (17)	0.61788 (11)	0.01314 (18)	
N1	0.9484 (2)	0.36700 (15)	0.44206 (10)	0.01379 (17)	
H01	1.135 (6)	0.377 (4)	0.440 (3)	0.031 (6)*	
N2	0.7891 (2)	0.17967 (15)	0.38503 (10)	0.01379 (17)	
H02	0.743 (6)	0.111 (4)	0.433 (2)	0.025 (6)*	
N3	0.7208 (2)	1.01131 (16)	0.59308 (10)	0.01420 (17)	
C3A	0.6136 (3)	1.11873 (18)	0.69578 (11)	0.01407 (18)	
C4	0.4492 (3)	1.2765 (2)	0.70032 (13)	0.0202 (2)	
H4	0.401227	1.318921	0.630063	0.024*	
C5	0.3581 (3)	1.3691 (2)	0.80960 (15)	0.0232 (3)	
H5	0.245566	1.475698	0.813913	0.028*	
C6	0.4294 (3)	1.3082 (2)	0.91385 (13)	0.0214 (2)	
H6	0.364372	1.374379	0.987636	0.026*	
C7	0.5928 (3)	1.1534 (2)	0.91122 (12)	0.0191 (2)	
H7	0.642508	1.113038	0.982118	0.023*	
C7A	0.6821 (3)	1.05845 (18)	0.80085 (11)	0.01465 (19)	
C8	1.0007 (3)	0.72931 (17)	0.52744 (11)	0.01505 (19)	
H8A	1.188694	0.709803	0.566030	0.018*	
H8B	1.035889	0.785089	0.459045	0.018*	
C9	0.8084 (3)	0.52723 (17)	0.47944 (11)	0.01345 (18)	
O1	0.5458 (2)	0.51401 (15)	0.47552 (10)	0.01766 (17)	
C10	0.6341 (3)	0.15543 (17)	0.27343 (11)	0.01340 (18)	
O2	0.6698 (2)	0.27818 (15)	0.21612 (10)	0.01878 (17)	
C11	0.4201 (3)	−0.03063 (17)	0.22693 (11)	0.01309 (18)	
C12	0.4296 (3)	−0.19751 (18)	0.27175 (12)	0.01574 (19)	
H12	0.576152	−0.195084	0.335201	0.019*	
C13	0.2252 (3)	−0.36724 (18)	0.22370 (12)	0.0172 (2)	
H13	0.233253	−0.481009	0.253846	0.021*	
C14	0.0089 (3)	−0.37052 (19)	0.13160 (12)	0.0180 (2)	
H14	−0.132276	−0.485758	0.099661	0.022*	
C15	−0.0006 (3)	−0.2051 (2)	0.08620 (13)	0.0191 (2)	
H15	−0.147919	−0.208041	0.022925	0.023*	
C16	0.2046 (3)	−0.03535 (19)	0.13308 (12)	0.0164 (2)	
H16	0.198429	0.076976	0.101456	0.020*	

### Atomic displacement parameters (Å^2^)

**Table:**

	*U*^11^	*U*^22^	*U*^33^	*U*^12^	*U*^13^	*U*^23^
S1	0.01905 (14)	0.01772 (12)	0.01655 (12)	0.00614 (9)	0.00163 (9)	0.00577 (9)
C2	0.0104 (5)	0.0131 (4)	0.0151 (4)	0.0011 (3)	0.0022 (3)	0.0026 (3)
N1	0.0089 (4)	0.0117 (4)	0.0194 (4)	0.0008 (3)	0.0019 (3)	0.0024 (3)
N2	0.0133 (4)	0.0114 (4)	0.0158 (4)	−0.0007 (3)	0.0011 (3)	0.0038 (3)
N3	0.0136 (4)	0.0139 (4)	0.0150 (4)	0.0025 (3)	0.0025 (3)	0.0034 (3)
C3A	0.0126 (5)	0.0129 (4)	0.0161 (4)	0.0021 (3)	0.0019 (3)	0.0030 (3)
C4	0.0209 (6)	0.0178 (5)	0.0229 (6)	0.0084 (4)	0.0036 (4)	0.0054 (4)
C5	0.0203 (6)	0.0198 (5)	0.0274 (6)	0.0077 (4)	0.0049 (5)	0.0008 (5)
C6	0.0173 (6)	0.0214 (6)	0.0212 (6)	0.0023 (4)	0.0051 (4)	−0.0023 (4)
C7	0.0182 (6)	0.0214 (5)	0.0156 (5)	0.0017 (4)	0.0032 (4)	0.0015 (4)
C7A	0.0132 (5)	0.0146 (4)	0.0152 (4)	0.0019 (3)	0.0023 (3)	0.0025 (3)
C8	0.0104 (5)	0.0126 (4)	0.0200 (5)	0.0006 (3)	0.0042 (4)	0.0006 (4)
C9	0.0106 (5)	0.0128 (4)	0.0160 (4)	0.0009 (3)	0.0026 (3)	0.0025 (3)
O1	0.0094 (4)	0.0163 (4)	0.0253 (4)	0.0008 (3)	0.0032 (3)	0.0026 (3)
C10	0.0128 (5)	0.0120 (4)	0.0158 (4)	0.0013 (3)	0.0032 (3)	0.0043 (3)
O2	0.0217 (5)	0.0158 (4)	0.0194 (4)	−0.0020 (3)	0.0015 (3)	0.0081 (3)
C11	0.0133 (5)	0.0113 (4)	0.0148 (4)	0.0006 (3)	0.0029 (3)	0.0039 (3)
C12	0.0158 (5)	0.0130 (4)	0.0182 (5)	0.0005 (4)	0.0007 (4)	0.0051 (4)
C13	0.0192 (6)	0.0121 (4)	0.0198 (5)	−0.0009 (4)	0.0029 (4)	0.0047 (4)
C14	0.0170 (5)	0.0156 (5)	0.0190 (5)	−0.0022 (4)	0.0028 (4)	0.0025 (4)
C15	0.0167 (6)	0.0193 (5)	0.0198 (5)	−0.0015 (4)	−0.0015 (4)	0.0058 (4)
C16	0.0158 (5)	0.0156 (5)	0.0179 (5)	0.0003 (4)	0.0005 (4)	0.0065 (4)

### Geometric parameters (Å, º)

**Table:**

S1—C7A	1.7310 (13)	C11—C16	1.3994 (17)
S1—C2	1.7422 (12)	C12—C13	1.3909 (17)
C2—N3	1.2993 (16)	C13—C14	1.390 (2)
C2—C8	1.4933 (17)	C14—C15	1.3911 (19)
N1—C9	1.3488 (16)	C15—C16	1.3919 (18)
N1—N2	1.3901 (14)	N1—H01	0.88 (3)
N2—C10	1.3747 (16)	N2—H02	0.85 (3)
N3—C3A	1.3939 (16)	C4—H4	0.9500
C3A—C4	1.4018 (18)	C5—H5	0.9500
C3A—C7A	1.4056 (17)	C6—H6	0.9500
C4—C5	1.386 (2)	C7—H7	0.9500
C5—C6	1.401 (2)	C8—H8A	0.9900
C6—C7	1.385 (2)	C8—H8B	0.9900
C7—C7A	1.3967 (19)	C12—H12	0.9500
C8—C9	1.5237 (16)	C13—H13	0.9500
C9—O1	1.2266 (15)	C14—H14	0.9500
C10—O2	1.2222 (14)	C15—H15	0.9500
C10—C11	1.4925 (16)	C16—H16	0.9500
C11—C12	1.3967 (17)		
			
C7A—S1—C2	89.49 (6)	C14—C15—C16	120.28 (12)
N3—C2—C8	124.45 (11)	C15—C16—C11	119.80 (11)
N3—C2—S1	115.83 (9)	C9—N1—H01	123.3 (19)
C8—C2—S1	119.70 (9)	N2—N1—H01	116.8 (19)
C9—N1—N2	119.00 (10)	C10—N2—H02	123.3 (18)
C10—N2—N1	117.28 (10)	N1—N2—H02	114.4 (18)
C2—N3—C3A	110.61 (10)	C5—C4—H4	120.7
N3—C3A—C4	125.26 (11)	C3A—C4—H4	120.7
N3—C3A—C7A	114.95 (11)	C4—C5—H5	119.4
C4—C3A—C7A	119.79 (12)	C6—C5—H5	119.4
C5—C4—C3A	118.52 (13)	C7—C6—H6	119.4
C4—C5—C6	121.11 (13)	C5—C6—H6	119.4
C7—C6—C5	121.18 (13)	C6—C7—H7	121.1
C6—C7—C7A	117.77 (13)	C7A—C7—H7	121.1
C7—C7A—C3A	121.63 (12)	C2—C8—H8A	109.5
C7—C7A—S1	129.24 (10)	C9—C8—H8A	109.5
C3A—C7A—S1	109.13 (9)	C2—C8—H8B	109.5
C2—C8—C9	110.93 (10)	C9—C8—H8B	109.5
O1—C9—N1	123.27 (11)	H8A—C8—H8B	108.0
O1—C9—C8	121.67 (11)	C13—C12—H12	119.9
N1—C9—C8	115.06 (10)	C11—C12—H12	119.9
O2—C10—N2	122.11 (11)	C14—C13—H13	120.0
O2—C10—C11	122.31 (11)	C12—C13—H13	120.0
N2—C10—C11	115.59 (10)	C13—C14—H14	120.0
C12—C11—C16	119.69 (11)	C15—C14—H14	120.0
C12—C11—C10	122.96 (11)	C14—C15—H15	119.9
C16—C11—C10	117.35 (10)	C16—C15—H15	119.9
C13—C12—C11	120.15 (11)	C15—C16—H16	120.1
C14—C13—C12	120.05 (11)	C11—C16—H16	120.1
C13—C14—C15	120.02 (11)		
			
C7A—S1—C2—N3	−0.19 (10)	N2—N1—C9—O1	6.66 (18)
C7A—S1—C2—C8	−178.46 (10)	N2—N1—C9—C8	−173.21 (10)
C9—N1—N2—C10	66.44 (15)	C2—C8—C9—O1	27.72 (16)
C8—C2—N3—C3A	178.23 (11)	C2—C8—C9—N1	−152.41 (11)
S1—C2—N3—C3A	0.05 (14)	N1—N2—C10—O2	12.34 (18)
C2—N3—C3A—C4	−179.93 (12)	N1—N2—C10—C11	−167.79 (10)
C2—N3—C3A—C7A	0.16 (15)	O2—C10—C11—C12	161.41 (13)
N3—C3A—C4—C5	−179.93 (13)	N2—C10—C11—C12	−18.46 (17)
C7A—C3A—C4—C5	0.0 (2)	O2—C10—C11—C16	−17.71 (18)
C3A—C4—C5—C6	0.4 (2)	N2—C10—C11—C16	162.42 (11)
C4—C5—C6—C7	−0.1 (2)	C16—C11—C12—C13	−0.29 (19)
C5—C6—C7—C7A	−0.6 (2)	C10—C11—C12—C13	−179.40 (12)
C6—C7—C7A—C3A	0.96 (19)	C11—C12—C13—C14	−0.6 (2)
C6—C7—C7A—S1	−179.59 (11)	C12—C13—C14—C15	0.9 (2)
N3—C3A—C7A—C7	179.25 (12)	C13—C14—C15—C16	−0.4 (2)
C4—C3A—C7A—C7	−0.65 (19)	C14—C15—C16—C11	−0.5 (2)
N3—C3A—C7A—S1	−0.30 (13)	C12—C11—C16—C15	0.84 (19)
C4—C3A—C7A—S1	179.80 (10)	C10—C11—C16—C15	179.99 (12)
C2—S1—C7A—C7	−179.25 (13)	O1—C9—N1—H01	175 (2)
C2—S1—C7A—C3A	0.26 (9)	O2—C10—N2—H02	166 (2)
N3—C2—C8—C9	−97.86 (14)	H01—N1—N2—H02	101 (3)
S1—C2—C8—C9	80.26 (12)		

### Hydrogen-bond geometry (Å, º)

**Table:**

*D*—H···*A*	*D*—H	H···*A*	*D*···*A*	*D*—H···*A*
N1—H01···O1^i^	0.88 (3)	2.02 (3)	2.8438 (14)	157 (3)
N2—H02···N3^ii^	0.85 (3)	2.15 (3)	2.9736 (15)	162 (3)

Symmetry codes: (i) *x*+1, *y*, *z*; (ii) *x*, *y*−1, *z*.
